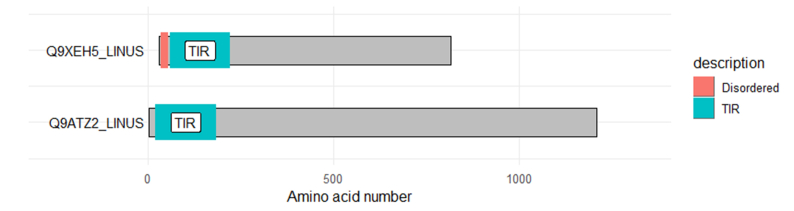# Diversity, abundance, and domain architecture of plant NLR proteins in *Fabaceae*

**DOI:** 10.1016/j.heliyon.2024.e34475

**Published:** 2024-07-12

**Authors:** Vishal Singh Negi, Rajagopalbabu Srinivasan, Bhabesh Dutta

**Affiliations:** a Department of Plant Pathology, University of Georgia, Tifton, GA, 31793, USA; b Florida Department of Agriculture and Consumer Services, Division of Plant Industry, Gainesville, FL, 32608, USA; c Department of Entomology, University of Georgia, Griffin, GA, 30223, USA

**Keywords:** NLR proteins, *Fabaceae*, Nucleotide-binding, Leucine-rich repeats, Resistance genes

## Abstract

The resistance (*R*) gene family in plants is a vital component of the plant defense system, enabling host resistance against pathogens through interactions with pathogen effector proteins. These R genes often encode nucleotide-binding (NB-ARC or N) and leucine-rich-repeat (LRR or L) domains, collectively forming the NLR protein family. The NLR proteins have been widely explored in crops from *Poaceae* and *Brassicaceae*, but limited studies are available for crops in other families, including *Fabaceae*. To address this gap, we conducted a comprehensive genome-wide analysis of putative NLR proteins in nine *Fabaceae* crops, including *Glycine max*, *Lupinus angustifolius*, *Medicago truncatula*, *Pisum sativum*, *Phaseolus vulgaris*, *Trifolium pratense*, *Vigna angularis*, *Vigna radiata*, and *Vigna unguiculata*. Our study revealed a substantial variation in the number of NLR proteins, independent of genome size. Notably, the NB-ARC domain exhibited a preferential co-occurrence with a specific LRR domain (IPR001611) in *Fabaceae*. Furthermore, through protein signature analysis, we identified both species-specific and shared domains across the nine crops. By classifying the identified proteins into seven distinct classes (N, L, CN, TN, NL, CNL, and TNL), we observed species-specific clustering within the CN, TN, and CNL classes, reflecting the diversification of species within *Fabaceae*. This genome-wide study enhances our understanding of the NLR protein repertoire and comprehensive protein signatures in nine *Fabaceae* species and provides valuable insights into plant defense mechanisms.

## 1 Introduction

Plants and animals possess defense mechanisms to protect themselves against pathogens. While animals have developed intricate immune systems, including innate and adaptive immunity, plants lack specialized immune cells and a circulatory system, limiting their ability to mount an adaptive immune response [1]. However, plants exhibit a robust innate immune response [2,3] against various pathogens, such as bacteria, viruses, nematodes, insects, and fungi. The plant innate immune system consists of pathogen-associated molecular patterns (PAMP)-triggered immunity (PTI) and effector-triggered immunity (ETI) [4]. PTI relies on cell surface receptors to recognize PAMPs, while ETI operates within plant cells, employing resistance proteins (R proteins) to sense pathogen invasion and initiate downstream signaling events leading to resistance responses. ETI involves a specific interplay between pathogen effector proteins encoded by avirulence genes (*avr*) and host plant R proteins. Pathogen resistance is established when the pathogen carries the corresponding *avr* gene and the host possesses the matching R gene. In the absence of either gene, the pathogen can cause disease in the plant [5,6] (see Table 4).

R proteins exhibit notable sequence and molecular functional divergence [1,[7], [8], [9], [10]]. Despite high divergence, the majority of the R proteins exhibit nucleotide-binding site and leucine-rich repeat (LRR or L) domains [4,6,11,12]. The nucleotide-binding site contains the NB-ARC domain (NB-ARC or N) belonging to the STAND (signal transduction ATPases with numerous domains) superfamily, which triggers signal transduction cascades leading to disease resistance [13]. The LRR domain facilitates protein-protein interaction [14] and plays an important role in the negative regulation of R protein; deletion or inactivation of the LRR domain results in constitutive expression of R protein [11].

In prior investigations, NLR genes have conventionally been characterized by the presence of the NBS domain. The categorization of NLRs into three subclasses—CNLs, TNLs, and RNLs—has been established through phylogenetic analysis focusing on the NBS domain. However, it is crucial to recognize that a substantial number of NLR proteins within each subclass exhibit a truncated domain architecture, resulting in the loss of N-terminal and/or C-terminal domains. Therefore, the truncated NLR proteins with N, L, NL, CN, and TN may belong to any of the three primary classes: CNL, TNL, or RNL. However, it is hard to guess, which of these three classes these truncated proteins belong to. Therefore, for clarity and convenience in this study, we have opted to retain them as distinct subclasses (N, NL, L, CN, TN) rather than grouping them within the three main classes. Proteins harboring both NB-ARC and LRR domains are referred to as NLR proteins in this study.

Given the expanding host range of pathogens and emerging crop diseases, research on plant defense and NLR genes is rapidly advancing. However, studies on NLR proteins have primarily focused on crops in *Poaceae* [15] and *Brassicaceae* [16,17]. The *Fabaceae* members contribute to ∼33 % of the protein in the human diet and 27 % of the world's crop production, making them the second-most economically significant family of food plants after the Poaceae [18]. Despite their economic significance, research on NLR proteins in *Fabaceae* is underrepresented in the literature. While some studies have touched upon a limited number of NLR proteins [[19], [20], [21], [22]], it is essential to acknowledge that comprehensive genome-wide analyses across diverse plant taxa, including Fabaceae, have been conducted in recent research endeavors [23], where authors have developed NLR atlas for over 300 angiosperm species. Another investigation into the evolution of NLR proteins in legumes illustrates a pattern of gain and loss in the interaction between microRNA and NBS genes throughout the course of legume evolution [24]. Previously, the integrated domains in NLR has been shown to play an important role in pathogen recognition [25,26]. However, majority of studies on NLR protein primarily emphasized evolutionary analysis and lacked a comprehensive examination of the domain architecture of NLR proteins. Therefore, in this study, we specifically focus on the domain architecture of NLR proteins in Fabaceae family. We screened the genomes of nine species of *Fabaceae*, including *Glycine* max (L.) Merr., *Phaseolus vulgaris* L., *Pisum sativum* L., *Vigna angularis* (Willd.) Ohwi & H. Ohashi, *Vigna radiata* (L.) R. Wilczek, *Vigna unguiculata* (L.) Walp., to identify the presence and distribution of NB-ARC and LRR domains. As the majority of characterized NLR proteins feature both NB-ARC and LRR domains [27,28], we assessed the co-occurrence of these domains in the sequences. Additionally, we analyzed the distribution of NB-ARC and LRR domains in each species and *Fabaceae* as a whole. Furthermore, we examined the protein sequences for complete InterPro domain profiles and performed classification and evolutionary analyses.

## 2 Materials and methods

### 2.1 Acquisition of reference R-genes

Resistance (R) genes encode R proteins that play crucial roles in pathogen resistance. These proteins are widely distributed in the plant kingdom, but their abundance varies across different species. Our primary aim was to examine the prevalence of established R genes, as documented in public databases, across diverse plant families, and to elucidate the domains present in the respective proteins. To accomplish this, we examined the pathogen receptor gene database (PRGdb; http://www.prgdb.org/prgdb4/), a publicly available curated database of reference R genes [29]. Information on known reference R-genes, including gene name, Uniprot ID, NCBI accession, R gene class, and species, was obtained from PRGdb in June 2022 and stored as a dataframe (S1a, Supplementary Materials S1). The data was then filtered for listed classes and species, and the taxonomic classification was obtained using the ‘tax_name’ function of the taxize package [30] as previously described [31,32]. RStudio [33] was used as the integrated development environment (IDE) for running all the R scripts and packages. Downstream processing of all the dataframes, such as data filtering, subsetting, etc., was performed using a R script. The reference R-gene dataframe (S1a, Supplementary Materials S1) was divided into separate dataframes for each R-gene class. The protein sequences for the corresponding Uniprot or GenBank accession for each class were pulled using a bash script. The Biostrings package [34] was used for the manipulation of fasta files. The protein sequences were then scanned against the InterPro database using the InterProScan package (InterProScan/5.55–88.0-foss-2019b) for Linux environment [35]. The InterProScan package utilizes various protein signature recognition methods to predict corresponding protein families, domains, and sites [35]. The InterProScan output files were processed to retrieve InterPro IDs for the reference R-genes using R [36]. Protein schematics, including identified domains for each class, were constructed using drawProteins [37] and ggplot2 [38] Bioconductor/R packages. The UpSetR [39] package was used for quantitative visualization of the distribution of InterPro domains in the 12 classes of reference R proteins and their intersections within each R protein class.

### 2.2 Retrieval of NLR genes and protein sequences from *Fabaceae*

NLR protein sequences and associated information were extracted using the biomaRt package [40,41], which offers an interface to connect with the Ensembl plant database for gene annotation and database mining. The list of available plant datasets in Ensembl was retrieved on September 22, 2022, using the ‘listDatasets’ function of the biomaRt library. All the species listed in the plant datasets were then classified into their respective families and genera using the taxize package [30]. Genes from *Fabaceae* plant datasets were mined using the getBM function of biomaRt. InterPro ids for the NB-ARC domain (IPR002182) and Leucine-rich repeat (LRR) domain (IPR001611, IPR011713, IPR013210, and IPR025875) were used as input values. The corresponding protein sequences, protein IDs, and transcript IDs, and their coordinates were extracted as dataframes for each species. The chord diagram for visualizing the relationship between the genome size and the number of NLR proteins and corresponding correlation analysis were performed using circlize [42] and smplot2 [43] packages. The NLR gene density on each chromosome of the nine species were plotted using ggplot2 package.

### 2.3 Distribution of NB-ARC and LRR domains in *Fabaceae*

The dataframes containing NLR protein sequences and associated details for each species were processed to remove duplicate sequences and retain only unique sequences. The distributions of the NB-ARC domain (IPR002182) and Leucine-Rich-Repeat (LRR) domains (IPR001611, IPR011713, IPR013210, and IPR025875) were calculated for each sequence in each species from the individual dataframes. The species-specific distributions of NB-ARC and LRR domains, as well as their individual occurrences and co-occurrences, were visualized quantitatively using the circlize package [42]. Additionally, the distribution of NB-ARC and LRR domains in the entire *Fabaceae* family was analyzed. The sequence counts of NB-ARC and LRR domains were pooled from individual dataframes and merged into one dataframe representing *Fabaceae*. The distribution, individual occurrence, and co-occurrence of NB-ARC and LRR domains in *Fabaceae* were visualized using the UpSetR package [39].

### 2.4 Protein domains in NLR proteins from *Fabaceae*

The unique sequences and their protein accessions were pooled from the processed dataframes of each species and converted into multifasta files using an R script as previously described [44]. These fasta files were then scanned against the InterPro database using the InterProScan package (InterProScan/5.55–88.0-foss-2019b). The output files from InterProScan were processed to retrieve InterPro IDs for the protein sequences from each species, which were saved as dataframes. The lists of total InterPro domains specific to each species were combined, and redundant InterPro domains were removed to obtain unique InterPro domains for each species. These individual lists of unique InterPro domains were merged and further filtered to eliminate any duplicate InterPro domains. The resulting list represents the unique InterPro domains found in putative NLR proteins of *Fabaceae*.

### 2.5 Classification of NLR proteins

Based on the presence or absence of the NB-ARC (N), Leucine-rich-repeat (L), coiled-coil domain (C), and Toll/IL receptor (T) domains in the protein sequences, the proteins were classified into seven primary classes: N, L, NL, CN, TN, CNL, and TNL. The InterProScan data from each species were initially divided into three protein groups: (i) N (proteins with NB-ARC but no Leucine-rich repeats), (ii) L (proteins with Leucine-rich repeats but no NB-ARC domains), and (iii) NL (proteins with both NB-ARC and Leucine-rich repeats). The N group was further separated into N (without C and T), CN, and TN, while the NL group was divided into NL (without C and T), CNL, and TNL.

### 2.6 Multiple sequence alignment and metric multidimensional scaling (MDS) analysis

The protein accessions were extracted for all seven classes (N, L, NL, CN, TN, CNL, and TNL) from each species. The corresponding protein sequences were pooled from the individual dataframes for each species, and new dataframes were created for each class, containing the protein accessions and sequences. These dataframes were then converted into multifasta files using an R script for further downstream analysis. For class-wise analysis of *Fabaceae*, the multifasta files from all the species were merged for each class so that each class represents sequences from all the species analyzed in this study. The multiple sequence alignment of protein sequences was performed using the msa package [45] in RStudio [33] as described previously [44]. The evolutionary distances of aligned sequences were calculated in the form of difference scores, which represent the proportion of sites that differ between two sequences [46]. The difference scores of aligned sequences were calculated using the bios2mds package [47]. The resultant difference scores were used to perform metric multidimensional analysis (MDS) using the cmdscale function of the R Stats package. The plots were created using the ggplot2 [38], gridextra, and ggpubr packages.

## 3 Results

### 3.1 Underrepresentation of *Fabaceae* in the public database of R genes

A total of 92 reference R genes were obtained from PRGdb (S1a, Supplementary Materials S1). These genes were categorized into 12 classes and found in 22 different plant species (S1b, Supplementary Materials S1). The distribution of 92 R genes among the classes was as follows: CN (n = 1), CNL (n = 11), LECRK (L-type lectin-domain-containing receptor-like kinases; n = 21), LYK (Lysin motif-containing receptor-like kinases; n = 14), LYP (Lysin motif-containing proteins; n = 6), N (n = 3), NL (n = 3), Other (n = 13), RLK (n = 9), RLP (n = 6), T (n = 2), and TNL (n = 3) (S1c, Supplementary Materials S1). Taxonomically, reference R genes from 22 species were associated with nine families: *Brassicaceae* (*n* = 37), *Solanaceae* (*n* = 14), *Cucurbitaceae* (*n* = 2), *Poaceae* (*n* = 29), *Malvaceae* (*n* = 2), *Linaceae* (*n* = 2), *Fabaceae* (*n* = 4), *Chenopodiaceae* (*n* = 1), and *Funariaceae* (*n* = 1) (Fig. 1). Notably, the representation of R genes in *Cucurbitaceae*, *Malvaceae*, *Linaceae, Fabaceae, Chenopodiaceae*, and *Funariaceae* was lower compared to *Brassicaceae*, *Poaceae*, and *Solanaceae*. In particular, *Fabaceae* was represented by only four R genes in the PRGdb, all belonging to the LYK class.

**Fig. 1:**
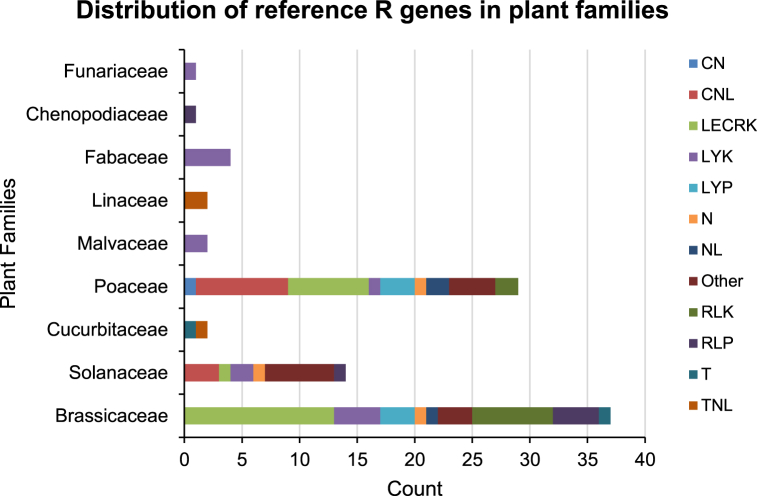
**Distribution of reference R gene classes in plant families.** The stacked bar plot displays the number of reference proteins for each class of R gene (CN, CNL, LECRK, LYK, LYP, N, NL, Other, RLK, RLP, T, and TNL) as well as the plant families in which they were identified. Each class is shown in different colors. (For interpretation of the references to color in this figure legend, the reader is referred to the Web version of this article.)

The protein sequences corresponding to Uniprot or GenBank accessions for each class (S2, Supplementary Materials) underwent InterPro database analysis to identify protein signatures. A total of 453 InterPro domains were identified, with varying distribution across classes: 2 in CN, 26 in CNL, 150 in LECRK, 75 in LYK, 31 in LYP, 10 in N, 9 in NL, 27 in Other, 67 in RLK, 36 in RLP, 13 in T, and 7 in TNL classes. (S3, Supplementary Materials). After eliminating duplicates within each class, a total of 60 unique InterPro domains were identified, distributed across CN (n = 2), CNL (n = 4), LECRK (n = 10), LYK (n = 7), LYP (n = 1), N (n = 3), NL (n = 5), Other (n = 8), RLK (n = 9), RLP (n = 4), T (n = 4), and TNL (n = 3) classes (S4, Supplementary Materials). Among these unique domains, 18 were exclusively present in a single class, while 12 were shared among two or more classes, resulting in a total of 30 distinct domains distributed across the 12 classes of R genes [Fig. 2A, B]. There were 30 distinct domains in total (IPR000157, IPR000858, IPR001220, IPR001480, IPR002182, IPR008808, IPR009743, IPR019825, IPR024788, IPR018392, IPR038005, IPR041118, IPR001611, IPR011713, IPR013210, IPR025875, IPR000719, IPR001245, IPR008266, IPR008271, IPR017441, IPR021820, IPR007110, IPR003609, IPR001781, IPR004316, IPR006936, IPR002913, IPR022087, and IPR045344) in all the reference R proteins.

**Fig. 2:**
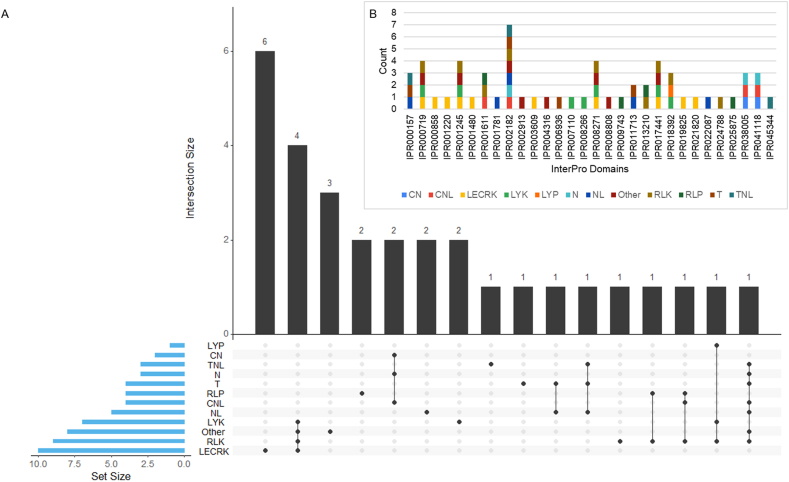
**Distribution of InterPro domains in 12 classes of reference R proteins.** (A) The UpSet plot shows the intersection of InterPro domains in R gene classes. The matrix layout for all intersections of 30 InterPro domains in 12 different R gene classes (CN, CNL, LECRK, LYK, LYP, N, NL, Other, RLK, RLP, T, and TNL) sorted by the set size. The presence and absence of domains in each class are shown by dark and light circles, respectively. (B) The stacked bar chart shows a representation of each InterPro domain in one or more R gene classes.

Protein schematics (S5, Supplementary Materials) showed that some protein sequences did not exhibit the expected domain architecture for their respective classes. For instance, the protein sequence with UniProt ID E3WF10 is classified in the CN class according to the PRGdb, but its protein schematic displays only the C domain (RX_N_CC_like), while the N domain (NB-ARC) is missing. In the CNL class, eight out of nine sequences did not possess a leucine-rich repeat region, which is characteristic of this class. On the other hand, the class N proteins displayed both the N domain (NB-ARC) and the C domain (RX.CC_like), suggesting that they should be reclassified into the CN class instead. Additionally, one sequence (UniProt ID Q9FKN7) in the NL class exhibited the presence of T domain (TIR), N domain (NB-ARC), and L domain (LIM zinc-binding), indicating that it should be classified in the TNL class instead.

### 3.2 Genome size independent NLR distribution in Fabaceae

Among the 30 unique domains identified in NLR genes from PRGdb, some domains (e.g.: protein kinase, serine-threonine kinase, tyrosine protein kinase, ATP binding site, etc.) are also found in non-R proteins. Including such domains in the mining of R proteins would also mine proteins that carry these domains but are not R proteins. Additionally, some domains, such as C-JID, PAN/Apple, and LIM zinc-binding, are part of a multidomain family and are present in R proteins along with other major domains such as NB-ARCC, RX_N_CC_like, leucine-rich repeat, LySM, TIR, etc. Hypothetically, including these major domains in the mining process would extract the multidomain R proteins, which would carry other complementary domains of the respective classes. Therefore, we decided against mining NLR genes on a class-by-class basis and instead used the InterPro domains for NB-ARC (IPR002182) and LRR (IPR001611, IPR011713, IPR013210, and IPR025875) as input keys without first specifying the class. At the time of analysis (Sept. 2022), Ensembl had 124 plant datasets (S6a, Supplementary Materials S6), which was then classified into their respective families and genera (S6b, Supplementary Materials S6). The dataframe containing species, datasets, genus, and family were then filtered into *Fabaceae*, which comprises reference genomes from nine species, including *Glycine max, Lupinus angustifolius, Medicago truncatula, Phaseolus vulgaris, Pisum sativum, Trifolium pratense, Vigna angularis, Vigna radiata,* and *Vigna unguiculate*. The taxonomic details and the version of genome assembly of these species are listed in Table S6c (Supplementary Materials S6).

A total of 9038 NLR proteins and 7829 corresponding genes were identified in nine representative species of *Fabaceae* (Table 1). However, *Fabaceae* family displayed wide variation in the count of these genes and proteins in the representative species. The highest number of NLR proteins (1756) and their respective genes were identified in *M. truncatula,* followed by *G.* max (1356 genes for 1725 NLR proteins), while *V. radiata* displayed the lowest number with 445 genes for 453 NLR proteins. It's important to note that the number of NLR proteins and the genes that encode them do not correlate with the genome size of the species under study (Fig. 3B). The genome size of *M. truncatula* (465 Mb) is less than half of that of *G.* max (1100 Mb); however, these plant species nearly contain the same number of NLR proteins (Table 1 and Fig. 3). Interestingly, *T. pratense* has the smallest genome (420 Mb) among these species but it displayed 1074 NLR proteins, while *P. sativum,* with the largest genome (4450 Mb), exhibited only 877 NLR proteins (Table 1 and Fig. 3). The complete detail, such as InterPro id, peptide sequence, ensemble peptide id, ensemble gene id, and chromosome name, of the extracted NLR proteins and their genes for each of the nine representative plant species in *Fabaceae* is given in Table S7 (Supplementary Materials). Considering that the quality of gene annotations of the genomes may also affect the number of identified NLRs in each genome assemblies, we also tested the possible correlation between the total number of annotated genes and count of NLR genes in each genome. While a weak negative correlation (r = −0.25) was observed, the p-value (0.525) suggests this correlation is not statistically significant (Fig. 3C). This indicates that the number of NLR genes is not necessarily dependent on the overall number of annotated genes in a genome assembly. We also analyzed the chromosomal distribution of NLR genes in 1 Mb window size of these 9 species. The majority of NLR genes were found to be clustered across the length of the chromosomes in each species (Fig. 4).

**Table 1:** Counts of genome size, proteins with NB-ARC and/or LRR domains, and their corresponding genes in nine plant species within *Fabaceae*.

Species	Common Name	Genome Size (Mb)	Total Number of Annotated genes	Count of NLR Genes	Count of NLR Proteins
*Glycine max*	Soybean	1100	89662	1356	1725
*Lupinus angustifolius*	Lupin	951	35179	488	488
*Medicago truncatula*	Barrel clover	500	59988	1540	1756
*Pisum sativum*	Pea	4450	57835	530	608
*Phaseolus vulgaris*	Common bean	587	33910	814	877
*Trifolium pratense*	Red clover	420	42485	1067	1074
*Vigna angularis*	Adzuki bean	538	34819	587	587
*Vigna radiata*	Mung bean	494	23977	445	453
*Vigna unguiculata*	Cowpea	640	54348	1002	1470
	Total			7829	9038

**Fig. 3:**
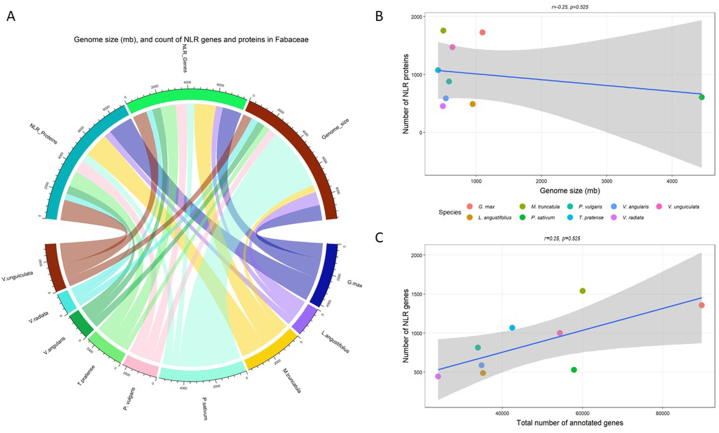
**Genome size (Mb), count of NLR proteins, and their corresponding genes in *Fabaceae*.** (A) The chord diagram represents the distribution of NB-ARC (N) and leucine-rich-repeat (L) domains containing proteins and genes in nine species of *Fabaceae*, including *Glycine max, Lupinus angustifolius, Medicago truncatula, Phaseolus vulgaris, Pisum sativum, Trifolium pratense, Vigna angularis, Vigna radiata*, and *Vigna unguiculata*. The genome size of each family is represented in megabase (mb). (B) The scatterplot represents the lack of correlation between genome size (Mb) and number of NLR proteins in each of the nine species. (C) The scatterplot shows lack of correlation between total number of annotated genes and total number of NLR identified in each genome.

**Fig. 4:**
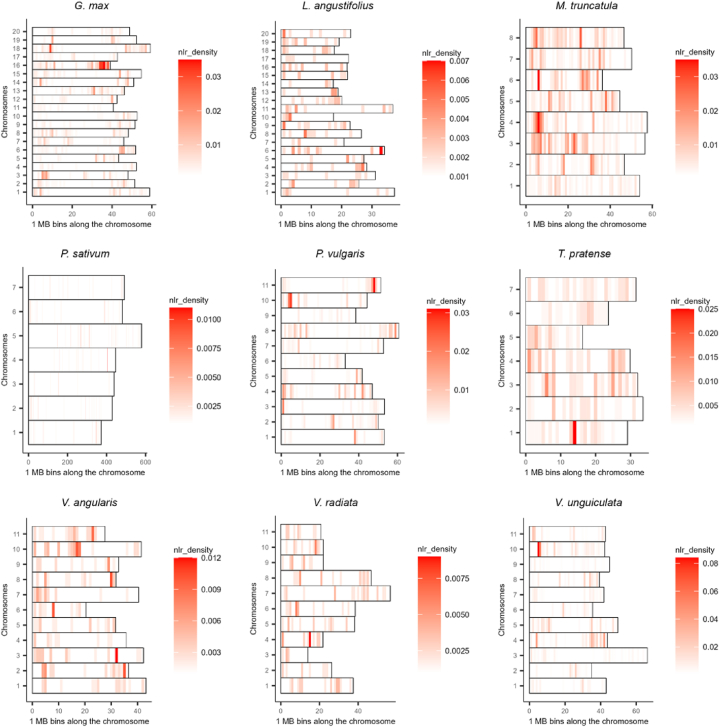
**Density plot of NLR genes within 1 Mb window size across the chromosomes of nine representative species of *Fabaceae*.** The horizontal axis represents the chromosome length in Mb, and the color gradient correspond to the density and distribution of NLR genes. (For interpretation of the references to color in this figure legend, the reader is referred to the Web version of this article.)

### 3.3 The NB-ARC (N) domain preferentially co-occur with IPR001611 in *Fabaceae*

The NB-ARC is represented by only one domain with one InterPro id, i.e., IPR002182, whereas Leucine-Rich-Repeat domains are represented by four different InterPro ids (IPR001611, IPR011713, IPR013210, and IPR025875). Therefore, our next goal was to examine if NB-ARC (N) has any preference for any particular LRR domain (L). With this goal, we screened the dataframe containing detailed information on NLR proteins identified from nine different species of Fabaceae for the individual occurrence and co-occurrences of N and L domains in 9038 R protein sequences. For the convenience of writing and data representation, the IPR002182, IPR001611, IPR011713, IPR013210, and IPR025875 domains will be indicated by N, L1, L2, L3, and L4, respectively.

In 93.4 % (8445) of the identified N and/or L domain-containing proteins, it was found that the L domains (L1, L2, L3, and L4) do not coexist with the N domain (Fig. 5). Individual N domain proteins (31.1 %, 2814), L1L3 domain proteins (28.4 %, 2569), and L1 domain proteins (24.1 %, 2178) make up the majority of these proteins (S8, Supplementary Materials). Individual occurrence of L1 (24.1 %) is remarkably higher than that of L2 (0.3 %), L3 (7 %) and L4 (0.1 %). The L4 domain largely coexists with other L3 and L4 domains as L1L4 (1.0 %, 87), L1L3L4 (0.8 %, 69), and L3L4 (0.4 %, 34). There are only eight (0.1 %) individual occurrences of L4, including two each in *G. max*, *T. pratense*, and *V. unguiculata*, and one each in *M. truncatula*, and *P. sativum*. We did not find any instance of L2 domain that coexists with L3 domain (L2L3) or L4 domain (L2L4) in the proteins where N and L domains do not coexist (Fig. 5). A total of 6.6 % (593) of the proteins displayed the coexistence of N and L domains (NL). The proteins with NL domains are predominated by NL1 (4.4 %, 400 proteins), and NL2 (1.6 %, 142 proteins). Proteins with NL1L2 and NL1L4 were represented by only 0.5 % (43 proteins), and 0.1 % (6 proteins), respectively (Fig. 5). Proteins with NL4 and NL2L4 domains were represented only once, suggesting their coexistence to be rare in *Fabaceae* (Fig. 5). Interestingly, none of the putative NLR proteins displayed coexistence of the N domain with L3 in any possible combination such as NL3, NL1L3, NL2L3, NL1L2L3, NL1L3L4, and NL2L3L4 in any of the 9038 proteins studied (Fig. 5).

**Fig. 5:**
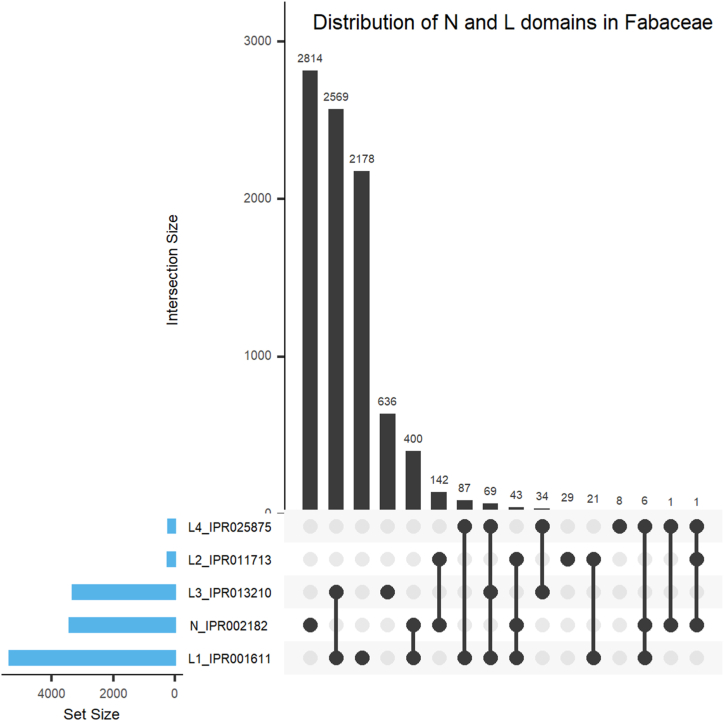
**Distribution of the NB-ARC (IPR002182) and Leucine-Rich-Repeat domains (IPR001611,** IPR011713**,** IPR013210**, and** IPR025875**) in proteins from *Fabaceae*.** The UpSet plot shows the intersection of NB-ARC (IPR002182) and Leucine-rich-Repeat (LRR) domains (IPR001611, IPR011713, IPR013210, and IPR025875) domains in proteins. The matrix layout for all intersections of these InterPro domains is sorted by set size. The light blue bar represents the set size (number of occurrences of a specific domain), whereas the dark gray bar shows the number of proteins displaying individual domains or specific domain combinations. The dark and light circles indicate sets that are included and not included, respectively, in the intersection. (For interpretation of the references to color in this figure legend, the reader is referred to the Web version of this article.)

### 3.4 Species-specific distribution of NB-ARC (N) and leucine-rich-repeats (L) domains

As different hosts respond differently against their invading pathogen or pathogen complex, it would be important to analyze the variation in the distribution of N and L (L1, L2, L3, and L4) domains in proteins from different plant species in *Fabaceae*. The species-specific distribution of N, L, and NL domains is shown in Fig. 6 and Table S8 (Supplementary Materials). In *Fabaceae*, N and L coexisted as NL1, NL2, NL1L2, NL1L4, NL4, and NL2L4. Among these NL-domain containing proteins (NLR proteins), NL1 appeared as the predominant protein, irrespective of the species. The coexistence of N and L1 (NL1) in *G. max, L. angustifolius, M. truncatula, P. vulgaris, P. sativum, T. pratense, V. angularis, V. radiata,* and *V. unguiculata* was found to be 68, 3, 88, 75, 15, 33, 30, 9, and 79 proteins, respectively. Importantly, NL1 in *P. vulgaris* represents 8.6 % (75 proteins) of its total NLR proteins, while it was found to be only 0.6 % (3 proteins) of the total NLR proteins in *L. angustifolius.*

**Fig. 6:**
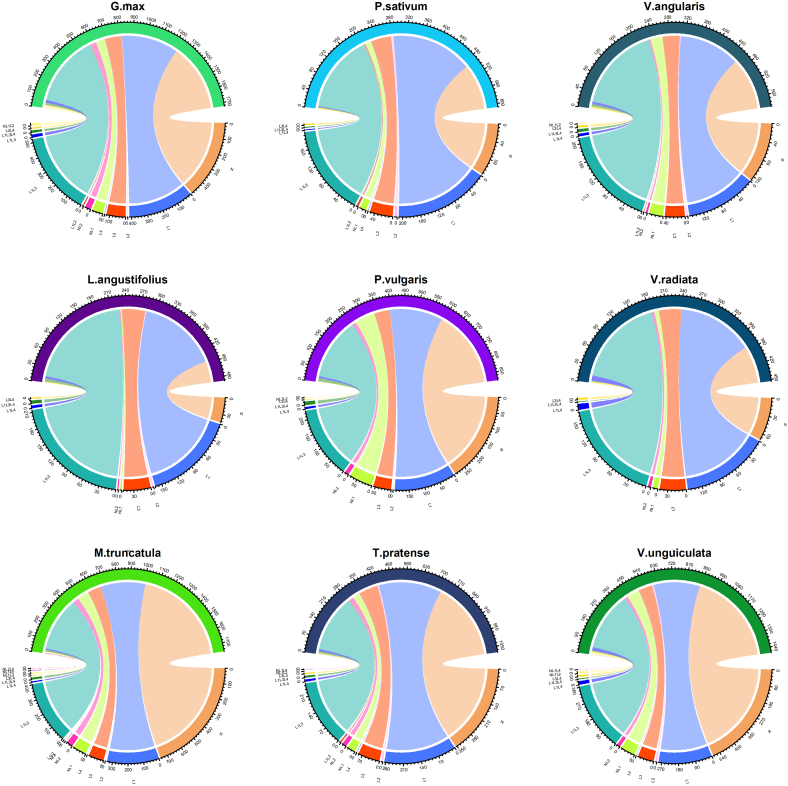
**Species-specific distribution of NB-ARC [IPR002182 (N)] and Leucine-rich-repeat domains [IPR001611 (L1),** IPR011713 **(L2),** IPR013210 **(L3),** IPR025875 **(L4) in proteins from nine different plant species belonging to *Fabaceae*.** The chord diagrams represent the occurrence of individual domains and the co-occurrence of two or more domains in NLR proteins identified in *Glycine max, Lupinus angustifolius, Medicago truncatula, Phaseolus vulgaris, Pisum sativum, Trifolium pratense, Vigna angularis, Vigna radiata*, and *Vigna unguiculata*.

The NL2 domain was identified as the second most prevalent NL-domain containing NLR proteins in *Fabaceae*. The NL2-domain containing proteins from *G.* max and *M. truncatula* represent 2.2 % (38 proteins) and 2.4 % (42 proteins) respectively, of their overall NLR proteins, which is higher than other species in *Fabaceae*. The NL2 prevalence holds true for all the species except *P. sativum*, which despite having the largest genome among the nine species studied, displayed no NL2-domain containing protein. The NL1L2 represents the third most prevalent combination of N and L domains in this family; however, this combination is absent in three species, including *L. angustifolius, P. sativum,* and *V. radiata*. In *G.* max and *V. unguiculata*, the NL1L2 makes up 0.8 % and 1 % of their total NLR proteins, respectively, which is noticeably higher than other species in *Fabaceae*.

The NL1L4, NL4, and NL2L4 were identified as the rare N and L combinations in the putative NLR proteins from *Fabaceae*. The NL1L2 combination of N and L domain was identified in six plant species in *Fabaceae*, including *G. max, M. truncatula, P. vulgaris, T. pratense, V. angularis,* and *V. unguiculata.* The NL4, and NL2L4 combinations of NL-domain-containing putative NLR proteins were found only in *M. truncatula* while NL1L4 was found in *M. truncatula*, *T. pratense*, and *V. unguiculata* only. It is noteworthy that only *M. truncatula* possesses all the combinations of N and L domains (NL1, NL2, NL1L2, NL1L4, NL4, and NL2L4) identified in *Fabaceae* (Fig. 6, and Table S8, Supplementary Materials).

### 3.5 Distribution of InterPro domains in NLR proteins from *Fabaceae* family

The sequence and corresponding details of NLR proteins from *Fabaceae* were extracted using NB-ARC and Leucine-Rich-Repeat domains as the input keys. Considering that the extracted sequence may also contain additional domains too, our next goal was to determine the domain signatures in all the NLR proteins from *Fabaceae*. In order to achieve this goal, the protein sequences (S2, Supplementary Materials) from all 9 species of *Fabaceae* were scanned against the InterPro database using the InterProScan to obtain all the InterPro domains in each sequence (S9, Supplementary Materials). A total of 4661, 1516, 4403, 1701, 2083, 2411, 1631, 1195, and 3740 InterPro domains were identified in the NLR proteins from *Glycine max, Lupinus angustifolius, Medicago truncatula, Pisum sativum, Phaseolus vulgaris, Trifolium pratense, Vigna angularis, Vigna radiata,* and *Vigna unguiculata*, respectively (Fig. 7). Although the counts of InterPro domains in NLR proteins of each species are in thousands, many of the domains were redundant. After removing duplicates, there were only 58, 49, 87, 61, 34, 84, 37, 44, and 42 unique InterPro domains in *Glycine max, Lupinus angustifolius, Medicago truncatula, Pisum sativum, Phaseolus vulgaris, Trifolium pratense, Vigna angularis, Vigna radiata,* and *Vigna unguiculata*, respectively (Fig. 7).

**Fig. 7:**
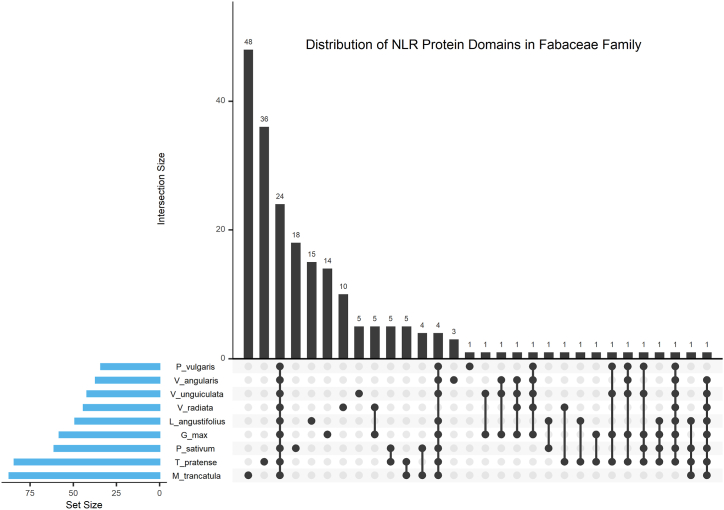
**The UpSet plot shows the distribution of InterPro domains found in the NLR proteins of 9 species from *Fabaceae*.** InterPro domains of all the NLR proteins from nine species, including *Glycine max, Lupinus angustifolius, Medicago truncatula, Phaseolus vulgaris, Pisum sativum, Trifolium pratense, Vigna angularis, Vigna radiata*, and *Vigna unguiculata,* were obtained. Redundant domains were excluded, and all the unique domains were plotted against nine sets representing nine species. The matrix layout for all intersections of unique InterPro domains was sorted by the set size. The light blue bar and the dark gray bar represent the set size (number of unique domains) of each species, and the number of domains intersecting across sets, respectively. The dark and light circles indicate sets that are included and not included, respectively, in the intersection. (For interpretation of the references to color in this figure legend, the reader is referred to the Web version of this article.)

The dataframe containing the unique InterPro domains from these 9 species (Table 2) was further filtered, and a total of 212 unique InterPro domains were identified in *Fabaceae*. The distribution of these 212 unique InterPro domains in these 9 species of *Fabaceae* is shown in Fig. 7. Out of 212, 11.3 % (24) of the InterPro domains were found to be present in all 9 species. A total of 48, 36, 18, 15, 14, 10, 5, 3, and 1 InterPro domains were found to be exclusively present in *M. truncatula, T. pratense, P. sativum*, *L. angustifolius*, *G. max, V. radiata*, *V. unguiculata, V. angularis,* and *P. vulgaris,* respectively (Fig. 7, Table 3, and S10, Supplementary Materials) (see Table 3).

**Table 2:** InterPro domains in NLR proteins from nine *Fabaceae* plant species.

Species	Count of			Unique InterPro Domain (Species)
	NLR Proteins	InterPro Domains	Unique Domains	
*Glycine max*	1725	4661	58	IPR001611, IPR008271, IPR000719, IPR013210, IPR001245, IPR017441, IPR024788, IPR025265, IPR041118, IPR002182, IPR038005, IPR000157, IPR011713, IPR045344, IPR008266, IPR021720, IPR006598, IPR025875, IPR001810, IPR041567, IPR003657, IPR044730, IPR002156, IPR019769, IPR020189, IPR008808, IPR044079, IPR000938, IPR007065, IPR000225, IPR026906, IPR026960, IPR001480, IPR003609, IPR000858, IPR002088, IPR041101, IPR045217, IPR002641, IPR013101, IPR045381, IPR000595, IPR003653, IPR001827, IPR001584, IPR032171, IPR035654, IPR013842, IPR031157, IPR000640, IPR000795, IPR025314, IPR000626, IPR007541, IPR002487, IPR001736, IPR004176, IPR013103
*Lupinus angustifolius*	488	1516	49	IPR000719, IPR001611, IPR001245, IPR002182, IPR013210, IPR041118, IPR008271, IPR017441, IPR045381, IPR021720, IPR041567, IPR001810, IPR025875, IPR000595, IPR008266, IPR008808, IPR024788, IPR038005, IPR006779, IPR007290, IPR000644, IPR025265, IPR022796, IPR040911, IPR019410, IPR045344, IPR002088, IPR032171, IPR000157, IPR011713, IPR000938, IPR044079, IPR002347, IPR020904, IPR003245, IPR041846, IPR005636, IPR000504, IPR034155, IPR041101, IPR017937, IPR013766, IPR021082, IPR045217, IPR000225, IPR002641, IPR000198, IPR000095, IPR000626
*Medicago truncatula*	1756	4403	87	IPR002182, IPR000157, IPR021720, IPR001611, IPR001245, IPR000719, IPR008271, IPR011713, IPR013210, IPR017441, IPR041118, IPR038005, IPR008266, IPR006121, IPR045344, IPR008615, IPR024788, IPR025875, IPR041101, IPR041567, IPR007111, IPR001810, IPR018181, IPR013126, IPR007810, IPR000547, IPR007679, IPR005630, IPR009810, IPR008808, IPR005636, IPR003439, IPR002715, IPR003656, IPR000668, IPR013201, IPR039417, IPR001781, IPR014039, IPR001969, IPR025265, IPR032171, IPR002843, IPR013525, IPR002933, IPR045381, IPR002641, IPR045217, IPR000225, IPR019775, IPR001680, IPR005134, IPR001296, IPR007529, IPR007009, IPR000368, IPR000626, IPR017930, IPR001005, IPR011545, IPR001650, IPR002464, IPR014001, IPR007502, IPR011709, IPR041588, IPR000070, IPR044079, IPR000938, IPR006747, IPR018526, IPR001892, IPR019189, IPR007645, IPR007646, IPR007644, IPR007641, IPR007642, IPR007120, IPR007647, IPR015712, IPR007121, IPR007877, IPR009450, IPR006671, IPR013763, IPR004367
*Pisum sativum*	608	1701	61	IPR017441, IPR000719, IPR013210, IPR008271, IPR001611, IPR002182, IPR001245, IPR001810, IPR041118, IPR038005, IPR024788, IPR000225, IPR002641, IPR045217, IPR013087, IPR000157, IPR011713, IPR021720, IPR001394, IPR018200, IPR041567, IPR041101, IPR007263, IPR008266, IPR044861, IPR026992, IPR005123, IPR044730, IPR000595, IPR005636, IPR008808, IPR045344, IPR004883, IPR004088, IPR025265, IPR025875, IPR003034, IPR045381, IPR002913, IPR003674, IPR024283, IPR044079, IPR000938, IPR018303, IPR002088, IPR041470, IPR001752, IPR018108, IPR001680, IPR019775, IPR000626, IPR006779, IPR014001, IPR000330, IPR004871, IPR041373, IPR041588, IPR006968, IPR032171, IPR003340, IPR001623
*Phaseolus vulgaris*	877	2083	34	IPR038005, IPR002182, IPR041118, IPR000157, IPR001611, IPR013210, IPR000719, IPR008271, IPR017441, IPR001245, IPR008808, IPR024788, IPR045344, IPR044730, IPR002156, IPR008266, IPR011713, IPR001810, IPR025875, IPR045217, IPR002641, IPR000225, IPR041567, IPR041101, IPR045381, IPR021720, IPR000626, IPR000938, IPR025265, IPR031691, IPR002088, IPR026906, IPR032171, IPR026960
*Trifolium pratense*	1074	2411	84	IPR008271, IPR017441, IPR000719, IPR013210, IPR001611, IPR002182, IPR001810, IPR001245, IPR041118, IPR025875, IPR015847, IPR001247, IPR038005, IPR011713, IPR032171, IPR024788, IPR000157, IPR003822, IPR008266, IPR045344, IPR005134, IPR024732, IPR024733, IPR024240, IPR021720, IPR000626, IPR002110, IPR026961, IPR007750, IPR044730, IPR002156, IPR013126, IPR018181, IPR025315, IPR009721, IPR004255, IPR021998, IPR008808, IPR045381, IPR000679, IPR025265, IPR041567, IPR013103, IPR039187, IPR026937, IPR044079, IPR000938, IPR006565, IPR012336, IPR045870, IPR013766, IPR002088, IPR008889, IPR008615, IPR002778, IPR005636, IPR028889, IPR018200, IPR001394, IPR026960, IPR041101, IPR013924, IPR006873, IPR000959, IPR003034, IPR016461, IPR001077, IPR001623, IPR002939, IPR013201, IPR001305, IPR018253, IPR000225, IPR045217, IPR002641, IPR000595, IPR000477, IPR025558, IPR005477, IPR006566, IPR018108, IPR018289, IPR018203, IPR024757
*Vigna angularis*	587	1631	37	IPR017441, IPR000719, IPR001611, IPR013210, IPR008271, IPR002182, IPR001245, IPR001810, IPR000626, IPR038005, IPR041118, IPR024788, IPR025875, IPR000938, IPR044079, IPR008266, IPR041567, IPR000157, IPR025265, IPR011713, IPR021720, IPR008808, IPR045381, IPR013101, IPR026960, IPR032171, IPR045344, IPR002088, IPR026906, IPR017907, IPR018957, IPR003657, IPR041101, IPR002048, IPR000225, IPR002641, IPR045217
*Vigna radiata*	453	1195	44	IPR002182, IPR001611 IPR041101, IPR041567, IPR008271, IPR013210, IPR017441, IPR000719, IPR001245, IPR003657, IPR008266, IPR025875, IPR041118, IPR038005, IPR008892, IPR000157, IPR028160, IPR035654, IPR000640, IPR031157, IPR000795, IPR013842, IPR001878, IPR011713, IPR045344, IPR024788, IPR008808, IPR025315, IPR021720, IPR001810, IPR044079, IPR000938, IPR025287, IPR008560, IPR001876, IPR045381, IPR026906, IPR000738, IPR002088, IPR025265, IPR002902, IPR000626, IPR010666, IPR001128
*Vigna unguiculata*	1470	3740	42	IPR001611, IPR013210, IPR000719, IPR021720, IPR008271, IPR017441, IPR001810, IPR002182, IPR041118, IPR008266, IPR000157, IPR038005, IPR001245, IPR041567, IPR025875, IPR011713, IPR024788, IPR004827, IPR045314, IPR045344, IPR002156, IPR044730, IPR006196, IPR003657, IPR013101, IPR045381, IPR008808, IPR032171, IPR002641, IPR000225, IPR045217, IPR026960, IPR044079, IPR000938, IPR041101, IPR001827, IPR026906, IPR000626, IPR041266, IPR002921, IPR025265, IPR002088

**Table 3:** Species-specific InterPro Domains in NLR proteins. The second and third columns show the count and the accession of the InterPro domain present exclusively in the species listed in column 1.

Species	Count InterPro domains	InterPro Domains and their description
*M. truncatula*	48	IPR007502 (Helicase-associated domain HA2), IPR011709 (DEAD-box helicase, OB fold), IPR000070 (Pectinesterase), IPR006747 (Protein of unknown function DUF599), IPR018526 (Glycoside hydrolase), IPR001892 (Ribosomal protein S13), IPR019189 (Ribosomal protein L27), IPR007645 (RNA polymerase Rpb2 domain 3), IPR007646 (RNA polymerase Rpb2 domain 4), IPR007644 (RNA polymerase, beta subunit), IPR007641 (RNA polymerase Rpb2, domain 7), IPR007642 (RNA polymerase Rpb2, domain 2), IPR007120 (DNA-directed RNA polymerase, subunit 2), IPR007647 (RNA polymerase Rpb2, domain 5), IPR015712 (DNA-directed RNA polymerase, subunit 2), IPR007121 (RNA polymerase, beta subunit, conserved site), IPR007877 (Protein of unknown function DUF707), IPR009450 (Phosphatidylinositol N-acetylglucosaminyltransferase subunit C), IPR006671 (Cyclin, N-terminal), IPR013763 (Cyclin-like), IPR004367 (Cyclin, C-terminal domain), IPR001296 (Glycosyl transferase, family 1), IPR007529 (Zinc finger, HIT-type), IPR007009 (Shq1 protein domain), IPR000368 (Sucrose synthase), IPR017930 (Myb domain), IPR001005 (SANT/Myb domain), IPR011545 (DEAD/DEAH box helicase domain), IPR001650 (Helicase, C-terminal), IPR002464 (DNA/RNA helicase, ATP-dependent, DEAH-box type, conserved site), IPR006121 (Heavy metal-associated domain, HMA), IPR007111 (NACHT nucleoside triphosphatase), IPR007810 (Pep3/Vps18/deep orange), IPR000547 (Clathrin, heavy chain/VPS, 7-fold repeat), IPR007679 (Domain of unknown function DUF569), IPR005630 (Terpene synthase, metal-binding domain), IPR009810 (Late nodulin), IPR003439 (ABC transporter-like, ATP-binding domain), IPR002715 (Nascent polypeptide-associated complex NAC domain), IPR003656 (Zinc finger, BED-type), IPR000668 (Peptidase C1A, papain C-terminal), IPR039417 (Papain-like cysteine endopeptidase), IPR001781 (Zinc finger, LIM-type), IPR014039 (Translation elongation factor EFTs/EF1B, dimerization), IPR001969 (Aspartic peptidase, active site), IPR002843 (ATPase, V0 complex, c/d subunit), IPR013525 (ABC-2 type transporter), IPR002933 (Peptidase M20)
*T. pratense*	36	IPR015847 (Exoribonuclease, phosphorolytic domain 2), IPR001247 (Exoribonuclease, phosphorolytic domain 1), IPR003822 (Paired amphipathic helix), IPR024732 (Alpha-N-acetylglucosaminidase, C-terminal), IPR024733 (Alpha-N-acetylglucosaminidase, tim-barrel domain), IPR024240 (Alpha-N-acetylglucosaminidase, N-terminal), IPR002110 (Ankyrin repeat), IPR026961 (PGG domain), IPR007750 (Protein of unknown function DUF674), IPR009721 (O-acyltransferase WSD1, C-terminal), IPR004255 (O-acyltransferase, WSD1-like, N-terminal), IPR021998 (Alfin, N-terminal), IPR000679 (Zinc finger, GATA-type), IPR039187 (Zinc finger, GATA-type), IPR026937 (Strawberry notch, helicase C domain), IPR006565 (Bromodomain associated domain), IPR012336 (Thioredoxin-like fold), IPR045870 (TryX and NRX, thioredoxin domain), IPR008889 (VQ), IPR002778 (Signal recognition particle, SRP19 subunit), IPR028889 (Ubiquitin specific protease domain), IPR013924 (Ribonuclease H2, subunit C), IPR006873 (Protein of unknown function DUF620), IPR000959 (POLO box domain), IPR016461 (O-methyltransferase COMT-type), IPR001077 (O-methyltransferase domain), IPR002939 (Chaperone DnaJ, C-terminal), IPR001305 (Heat shock protein DnaJ, cysteine-rich domain), IPR018253 (DnaJ domain, conserved site), IPR000477 (Reverse transcriptase domain), IPR025558 (Domain of unknown function DUF4283), IPR005477 (Deoxyxylulose-5-phosphate synthase), IPR006566 (FBD domain), IPR018289 (MULE transposase domain), IPR018203 (GDP dissociation inhibitor), IPR024757 (Cell division protein FtsZ, C-terminal)
*P. sativum*	18	IPR013087 (Zinc finger C2H2-type), IPR007263 (DCC1-like thiol-disulfide oxidoreductase family), IPR044861 (Isopenicillin N synthase-like, Fe(2+) 2OG dioxygenase domain), IPR026992 (Non-haem dioxygenase N-terminal domain), IPR005123 (Oxoglutarate/iron-dependent dioxygenase), IPR004883 (Lateral organ boundaries, LOB), IPR004088 (K Homology domain, type 1), IPR002913 (START domain), IPR003674 (Oligosaccharyl transferase, STT3 subunit), IPR024283 (Translocase of chloroplast 159/132, membrane anchor domain), IPR018303 (P-type ATPase, phosphorylation site), IPR041470 (Gamma tubulin complex component protein, N-terminal), IPR001752 (Kinesin motor domain), IPR000330 (SNF2, N-terminal), IPR004871 (Cleavage/polyadenylation specificity factor, A subunit, C-terminal), IPR041373 (Reverse transcriptase, RNase H-like domain), IPR006968 (Root UVB sensitive family), IPR003340 (B3 DNA binding domain)
*L. angustifolius*	15	IPR007290 (Arv1 protein), IPR000644 (CBS domain), IPR022796 (Chlorophyll A-B binding protein), IPR040911 (Exostosin, GT47 domain), IPR019410 (Lysine methyltransferase), IPR002347 (Short-chain dehydrogenase/reductase SDR), IPR020904 (Short-chain dehydrogenase/reductase, conserved site), IPR003245 (Phytocyanin domain), IPR041846 (Early nodulin-like protein domain), IPR000504 (RNA recognition motif domain), IPR034155 (HRP1, RNA recognition motif 2), IPR017937 (Thioredoxin, conserved site), IPR021082 (Protein GAPT), IPR000198 (Rho GTPase-activating protein domain), IPR000095 (CRIB domain)
*G. max*	14	IPR006598 (Glycosyl transferase CAP10 domain), IPR019769 (Translation elongation factor, IF5A, hypusine site), IPR020189 (Translation elongation factor, IF5A C-terminal), IPR007065 (HPP), IPR001480 (Bulb-type lectin domain), IPR003609 (PAN/Apple domain), IPR000858 (S-locus glycoprotein domain), IPR003653 (Ulp1 protease family, C-terminal catalytic domain), IPR001584 (Integrase, catalytic core), IPR025314 (Domain of unknown function DUF4219), IPR007541 (Uncharacterised protein family, basic secretory protein), IPR002487 (Transcription factor, K-box), IPR001736 (Phospholipase D/Transphosphatidylase), IPR004176 (Clp, repeat (R) domain)
*V. radiata*	10	IPR008892 (Cold-regulated 413 protein), IPR028160 (Ribosome biogenesis protein Slx9-like), IPR001878 (Zinc finger, CCHC-type), IPR025287 (Wall-associated receptor kinase, galacturonan-binding domain), IPR008560 (Protein of unknown function DUF842), IPR001876 (Zinc finger, RanBP2-type), IPR000738 (WHEP-TRS domain), IPR002902 (Gnk2-homologous domain), IPR010666 (Zinc finger, GRF-type), IPR001128 (Cytochrome P450)
*V. unguiculata*	5	IPR004827 (Basic-leucine zipper domain), IPR045314 (G-box binding factor 1-like, bZIP domain, plant), IPR006196 (RNA-binding domain, S1, IF1 type), IPR041266 (EDS1, EP domain), IPR002921 (Fungal lipase-like domain)
*V. angularis*	3	IPR017907 (Zinc finger, RING-type, conserved site), IPR018957 (Zinc finger, C3HC4 RING-type), IPR002048 (EF-hand domain)
*P. vulgaris*	1	IPR031691 (Lipoyl synthase, N-terminal)

**Table 4:** Distribution of the N, L, and NL classes and their respective subclasses of NLR proteins in nine representative species of *Fabaceae*.

Species	Classes and subclasses						
	L	N			NL		
		N	CN	TN	NL	CNL	TNL
*Glycine max*	1101	171	146	175	44	27	53
*Lupinus angustifolius*	437	22	18	4	2	2	1
*Medicago truncatula*	863	233	220	292	39	39	67
*Pisum sativum*	468	51	54	10	8	7	0
*Phaseolus vulgaris*	476	113	138	82	13	15	15
*Trifolium pratense*	666	173	114	65	29	11	13
*Vigna angularis*	422	41	67	20	8	17	10
*Vigna radiata*	369	42	17	11	7	0	7
*Vigna unguiculata*	777	191	148	239	46	34	31

### 3.6 Distribution of NLR proteins from *Fabaceae* in different classes

We identified a total of 212 unique InterPro domains in NLR proteins from *Fabaceae*. For studying the distribution of putative NLR proteins in *Fabaceae*, we initially classify them into three major categories: (i) N (proteins with NB-ARC but no LRR domains), (ii) L (proteins with LRR but no NB-ARC domains), and (iii) NL (proteins with both NB-ARC and LRR domains). The N was further subdivided into three categories: N, CN, and TN, where C and T represent the N-terminus coiled-coil (CC) domain and Toll/IL receptor (TIR) domain, respectively. Similarly, the NL domain containing proteins were categorized into three classes: (i) NL (with no T or C domains), (ii) TNL (TIR-NB-ARC-L), and (ii) CNL (CC-NB-ARC-L).

Among N, L, and NL, the L was found to be the most abundant class in all the species, with 63.8 %, 49.2 %, 78 %, 56 %, 62 %, 90 %, 72 %, 81 %, and 53 % of total NLR proteins from *G. max, M. truncatula, P. sativum, P. vulgaris, T. pratense, L. angustifolius, V. angularis, V. radiata*, and *V. unguiculata*, respectively. None of the proteins in the L category display cooccurrence of L and C domains (CL) in any of the species except *P. sativum*, which exhibited 0.16 % of the total NLR proteins as CL subfamily (Table 4 and S11, Supplementary Materials). Only a small fraction (ranging from 0.05 % to 1.32 % of total NLR proteins) of L category exhibited the cooccurrence of T and L domains (TL). Interestingly, the L class of NLR proteins from *P. vulgaris* and *V. radiata* lacks both CL and TL subfamilies (Table 4 and S11, Supplementary Materials).

The N category appeared as the second most abundant group in NLR proteins with 29 %, 42.5 %, 19 %, 39 %, 33 %, 9 %, 22 %, 15 %, and 39 % of total NLR proteins from *G. max, M. truncatula, P. sativum, P. vulgaris, T. pratense, L. angustifolius, V. angularis, V. radiata*, and *V. unguiculata*, respectively. In all 9 species, the proteins in the N category primarily occurred as N (with no C and T domains), CN, and TN subfamilies (Table 4 and S11, Supplementary Materials). The occurrence of the CN subclass was found to predominate over the count of TN subclass in *L. angustifolius, P. sativum, P. vulgaris, T. pratense, V. angularis,* and *V. radiata*, while *G. max, M. truncatula*, and *V. unguiculata* displayed more TN subclass than CN subclass (Table 4 and S11, Supplementary Materials).

The NL category, representing putative NLR proteins, was found to be the least abundant class, ranging from 1 % to 8 % of the total NLR proteins (Table 4 and S11, Supplementary Materials). The NL class was categorized as NL (with no T or C domains), CNL, and TNL subclasses. Among the NL, CNL, and TNL subclasses, the NL subclass predominates in *P. sativum, T. pratense*, and *V. unguiculata*; the CNL subclass predominates in *V. angularis*; and the TNL subclass is more prevalent in *G. max,* and *M. truncatula*. The occurrence of CNL and TNL subclasses, NL and CNL subclasses, and NL and TNL subclasses were equally predominating in *P. vulgaris, L. angustifolius*, and *V. radiata*, respectively. Interestingly, *V. radiata and P. sativum* did not display any members of the CNL and TNL subfamilies of putative NLR proteins, respectively (Table 4 and S11, Supplementary Materials).

### 3.7 MDS analysis reveals species-specific clustering in CN, TN, and CNL classes of NLR proteins

We categorized the protein sequences into three major categories: N, L, and NL, where NL represents the putative NLR proteins. The N and L were classified into 4 classes, including N, CN, TN, and L, whereas the NL were classified into three classes including, NL, CNL, and TNL. This classification is based on the presence/absence of N, L, C, and T domains. However, these proteins displayed varying numbers of several other InterPro domains. This adds complexity to the architecture of each class and therefore, the construction of phylogenetic trees for these seven classes of proteins would not be a viable choice for comparative analysis. For our datasets, metric multidimensional scaling (MDS), would be a more suitable method for understanding the relationships among these proteins within each class in each species and in *Fabaceae* as a whole. MDS is an exploratory multivariate method to determine patterns in a distance matrix [48,49]. For species-wise MDS analysis, all the protein sequences from each species were first separated into their respective classes (N, L, NL, CN, TN, CNL, and TNL), followed by multiple sequence alignment using MUSCLE [50]. The matrix of pairwise distance was computed from the aligned sequences, and MDS analysis was performed from the resultant distance matrix. The proteins in class N were found to form three or more clusters in all nine species (S12, Supplementary Materials). The class L proteins in *V. radiata, T. pratense,* and *L. angustifolius* were mostly dispersed, while other species displayed indistinct clusters of class L proteins. Compared to other classes, TN and CN classes displayed more distinct clusters across all the species. In CN class, *G. max, M. truncatula, P. vulgaris,* and *T. pratense* displayed predominantly distinct clusters while in the TN class *V. unguiculata* exhibited discrete clusters. The count of NL, CNL, and TNL proteins in each species is small, and therefore, it is difficult to observe discrete clusters; however, these classes appear to form clustering patterns (S12, Supplementary Materials). Pooling these classes from all nine species and analyzing them using MDS analysis may potentially give a better understanding of their clustering pattern.

We aggregated each individual class from each species (aside from L) in order to represent all 9 species collectively for analyzing the class-wise relationships of these proteins across *Fabaceae*. Regardless of the species examined, class L was largely dispersed with no distinguishable clusters, hence we did not include it in the class-wise MDS analysis. The pooling of class-wise proteins from all the species resulted in the 1037 N, 196 NL, 922 CN, 898 TN, 152 CNL, and 197 TNL classes of NLR proteins from the *Fabaceae*. The N class of proteins appeared as six discrete clusters, and these clusters represented all nine species (Fig. 8). The clusters in the NL class of proteins were not sharp and aggregated, and like the clusters in N, the NL clusters were also represented by all 9 species. The class CN produced 6 sharp and aggregated clusters. Remarkably, one of the CN clusters (cluster 1, encircled with red dotted lines in Fig. 8) is predominately represented by *P. vulgaris*. Cluster 1 contains some proteins from the species *G. max, V. unguiculata*, and *V. angularis*, but none from the species *M. truncatula, L. angustifolius, P. sativum, V. radiata, or T. pratense.* The TN class of proteins also formed 3 discrete clusters that displayed species-specific representation (clusters 2, 3, and 4, encircled with red dotted lines in Fig. 8). Cluster 2 is predominated by proteins from *V. unguiculata,* followed by proteins from *P. vulgaris* and *V. angularis*, while the cluster lacks proteins from *G. max*, *L. angustifolius, M. truncatula, P. sativum, T. pratense*, and *V. radiata.* Cluster 3 is predominated by *M. truncatula*, and it is devoid of proteins from *P. vulgaris, V. angularis,* and *V. radiata*. Cluster 4 is largely represented by proteins from *G. max,* and *M. truncatula*, and it lacks proteins from *P. vulgaris* (Fig. 8). The CNL class appeared as five small but distinct clusters. One of the clusters (represented as cluster 5 in Fig. 8) is predominated by proteins from *V. radiata, G. max*, and *V. angularis,* while the cluster is completely devoid of proteins from *M. truncatula*, *L. angustifolius, P. sativum, T. pratense,* and *V. unguiculata*. Another cluster, labeled cluster 6 (Fig. 8), is dominated by proteins from *M. truncatula,* followed by *T. pratense,* and *V. radiata*. There were no proteins from other species in this cluster. The TNL class of proteins appeared as five discrete clusters. We did not observe species-specific distributions of proteins in TNL clusters (Fig. 8).

**Fig. 8:**
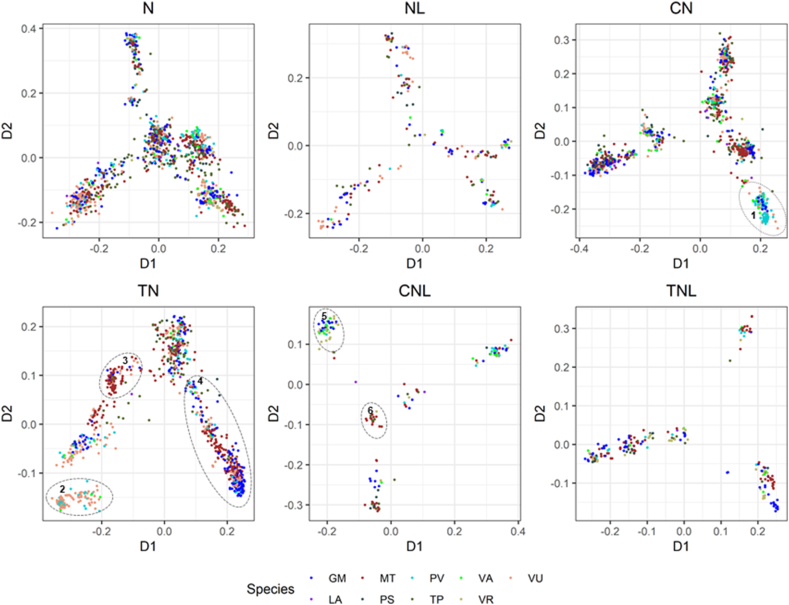
**Class-wise MDS representation of NLR proteins from nine different species of *Fabaceae*.** Each individual class, including N, NL, CN, TN, CNL, and TNL, were pooled from 9 species (GM: *Glycine max*, LA: *Lupinus angustifolius*, MT: *Medicago truncatula*, PS: *Pisum sativum*, PV: *Phaseolus vulgaris*, TP: *Trifolium pratense*, VA: *Vigna angularis*, VR: *Vigna radiata*, and VU: *Vigna unguiculata*) and their multiple alignments were obtained using MUSCLE. Their corresponding distance matrices were plotted in a low dimensional space by metric multidimensional scaling (MDS). Each species is represented with distinct colors. Important clusters are enclosed with dotted elliptic. (For interpretation of the references to color in this figure legend, the reader is referred to the Web version of this article.)

## 4 Discussion

*R* genes in the plant kingdom exist as rapidly evolving large gene families that are indispensable players in plant defense [14,51,52]. The public database for known *R* genes (PRGdb) represents 22 plant species distributed in 9 families. The taxonomic distribution of known *R* genes in PRGdb is predominated by the Brassicaceae, Poaceae, and Solanaceae families. In this study, we performed a genome-wide analysis to identify putative R protein repertoire in 9 species from *Fabaceae*.

Contrary to the general expectation that larger genomes would contain a higher number of genes [53], we did not observe a correlation between genome size and the abundance of NLR proteins in *Fabaceae*. For example, *Pisum sativum,* which has the largest genome (4450 Mb) among the 9 species analyzed, carries only 608 putative NLR proteins, while *M. truncatula*, with a small genome (500 Mb), harbors the highest number of NLR proteins (*n* = 1756). The number of NLR genes and proteins is comparable between *G.* max and *M. truncatula*, although *G.* max has a genome that is more than twice the size of *M. truncatula* genome. *G.* max is a palaeopolyploid [54,55] as opposed to the diploid *M. truncatula* [56,57]. Also, we did not observe any significant correlation between the total number of annotated genes and total number of NLR genes, suggesting that the count of NLR genes is not necessarily dependent on the overall number of annotated genes in a genome assembly. Additional dynamics, such as genome duplication events, gene silencing or deletion, as well as the expansion and tandem duplication of genes, may also significantly contribute to the count of NLRs in a genome. The functional redundancy due to genome duplication in polyploids often leads to silencing or deletion of genes from the duplicated genomes [54,58]. This could be a possible reason why the genome size of palaeopolyploid *G.* max is more than twice the genome size of *M. truncatula* while it harbors a comparable number of NLR proteins. Our conclusion of lack of correlation between 10.13039/100010156NLR genes and the genome size is further supported by a recent study where 10.13039/100010156NLR copy numbers were found to differ up to 66-fold among closely related species as a result of rapid gene loss and gain [23]. Moreover, in an evolutionary study on NLR genes, genes with the NB-ARC domain were found to display deletions or significant expansions with tandem duplications, resulting in the creation of many novel NLR genes [24]. Thus, the differential expansion of NLR genes in different species, regardless of genome size, may explain the lack of correlation between genome size and the number of NLR proteins.

A significant number of R proteins exhibit the co-occurrence of the NB-ARC (N) domain and the LRR motif [13,59]. LRR motifs, crucial for diverse protein-protein interactions due to their structural role [60], are found in proteins with varied functions [61]. Unlike the NB-ARC, the LRR exists in four types represented by InterPro ids: IPR001611 (L1), IPR011713 (L2), IPR013210 (L3), and IPR025875 (L4). We expect functionally related proteins with LRR domains to share the same LRR type. Among the proteins with N and/or L domains, only 6.6 % (*n* = 593) proteins displayed the coexistence of N and L. Within this group, 400 proteins displayed the coexistence of N with L1 (IPR001611), 142 proteins were found to carry N with L2 (IPR011713), and 43 existed as N with both L1 and L2. Even in the species-wise distribution of N and L domains, NL1 dominated over NL2. This indicates that NL1, NL2, and NL1L2 are the three major groups of NLR proteins in *Fabaceae*, which is further ascertained by the three major clusters that appeared in the MDS analysis of the NL class of NLR proteins. The L4 domain co-occurred with N in only two instances, while L3 never appeared with N. Considering that LRR motifs may occur in unrelated proteins [61], it is plausible that L3 domain-containing proteins serve roles other than defense resistance, though experimental validation is needed.

Regardless of the species and family, NLR proteins serve the same general purpose, which is to defend plants from invasive pathogens. However, NLR proteins display high diversity in their sequence, molecular function, protein architecture, and abundance [1,[7], [8], [9], [10]]. We identified 30 distinct domains in known R proteins from PRGdb, which not only points to the complex architecture of R proteins but also suggests a variety of molecular functions for these proteins. Also, it is noteworthy that the R proteins in the PRGdb only contain known R proteins and do not include unknown R proteins. Therefore, the 30 unique domains, identified in known R proteins, are not a true representation of the actual complexity of the architecture of NLR proteins. The species-specific complexity in the domain architecture of NLR proteins could not be deduced from the known R proteins contained in PRGdb due to the fact that the known R proteins from any given species reflect only a small portion of the actual NLR proteins from that species. Additionally, the real number of NLR proteins in any species may be significantly higher than the number of known R proteins in PRGdb. Therefore, it is conceivable that the number of domains may remarkably increase if undiscovered NLR proteins are considered.

Animals, unlike plants, possess specialized immune cells (B cells and T cells) that undergo genetic rearrangement to generate huge diversity in antibodies and T cell receptors [62,63]. Such enormous diversity helps them achieve a robust immune response against a vast range of pathogens. Despite lacking specialized immune cells, plants may be able to fight off insects or pathogens that have undergone virulence variation because of the allelic diversity in NLR genes [64,65]. Additionally, plant NLR proteins display high sequence divergence along with variation in their domain architecture. Hypothetically, such complexity in the architecture of NLR proteins would lead to diverse molecular functions, which in turn, offers an advantage in fighting against a diverse range of diseases. In our genome-wide analysis, we identified species-specific complexity in the domain architecture of NLR proteins. When NLR protein domains of *Fabaceae* species were examined, we observed noticeable variations in the number of unique domains in *Glycine* max (*n* = 58)*, Lupinus angustifolius* (*n* = 49)*, Medicago truncatula* (*n* = 87)*, Pisum sativum* (*n* = 61)*, Phaseolus vulgaris* (*n* = 34)*, Trifolium pratense* (*n* = 84)*, Vigna angularis* (*n* = 37)*, Vigna radiata* (*n* = 44)*,* and *Vigna unguiculata* (*n* = 42). The diversity in plant *NLR* gene families is suggested as an outcome of host and pathogen co-evolution under diverse environmental conditions [27]. As pathogens vary in their host range, it is not surprising to observe a large difference in the count of unique domains in NLR proteins in these plant species. In the nine species of *Fabaceae* screened, the number of unique domains spans from 34 (lowest count) in *P. vulgaris* to 87 (highest count) in *M. truncatula*. However, the count of unique domains in the nine species of *Fabaceae* was found to be 212, which is 2.4-fold higher than the count of unique domains in *M. truncatula.* This suggests that a large set of domains are species-specific, which was further confirmed through the intersection analysis of each domain in different species. Among 212 unique domains, only 24 were found to be shared by all nine species, while the rest of the unique domains displayed species-specific distribution. Remarkably, NLR proteins from *M. truncatula* and *T. pratense* alone were found to carry 48 and 36 unique domains, respectively, which are not present in any other species analyzed in this study, indicating that NLR proteins from both plant species possess a more complex architecture than those from other species in *Fabaceae* family. In contrast, the NLR protein architecture in *P. vulgaris, V. radiata, V. unguiculata*, and *V. angularis* appeared less complex. We discovered that only one domain is exclusive to *P. vulgaris*. Similarly, the exclusive domain counts for *V. radiata, V. unguiculata*, and *V. angularis* are 10, 5, and 3, respectively. Since complexity in the domain architecture of NLR proteins may potentially broaden the range of molecular functions that any species' NLR repertoire can perform, we hypothesize that a species with a more intricate NLR protein architecture, like *M. truncatula*, should be better equipped to fight against a range of plant pathogens than a species with a less intricate NLR protein architecture, like *P. vulgaris* or *P. sativum*. In general, weeds exhibit greater resilience to a wide range of plant pathogens than commercially grown crops [66]. *M. truncatula*, which is a small annual weedy legume species, was found to display resistance to major pathogens of *P. sativum* and *M. sativa* [67]. Another study demonstrated that broad-spectrum resistance to anthracnose is conferred on *M. sativa* by transgenic expression of the TNL class of NLR gene (RCT1 gene) from *M. truncatula* [68]. In our study, *M. truncatula* displayed the maximum number of unique domains in NLR proteins among all nine species investigated, which, along with the previous studies [67,68], supports our hypothesis of complex NLR protein architecture. Comprehensive and comparative studies focusing on the pathogen resistance responses of plant species with either higher (e.g., *M. truncatula*) or lower (e.g., *P. vulgaris*) complexity of NLR protein architecture against a wide range of plant pathogens (viruses, bacteria, fungi, nematodes, etc.) would be more insightful.

A previous study on NLR proteins from *Helianthus annuus* L. (sunflower) and *Arabidopsis thaliana* (L.) Heynh. (Arabidopsis) has reported species-specific clustering of CNL and TNL classes of NLR proteins [69]. We also observed species-specific clustering in the CN, TN, and CNL classes in the class-wise MDS analysis. A CN class cluster (cluster 1) is dominated by NLR proteins from *P. vulgaris,* followed by *G. max,* but is devoid of NLR proteins from *M. truncatula, T. pratense* and *P. sativum* Similarly, the TN class showed species-specific distribution, with cluster 2 dominated by NLR proteins from *V. unguiculata* and *P vulgaris,* and cluster 3 dominated by NLR proteins from *M. truncatula*. The CNL class of NLR proteins also showed clustering of NLR proteins from *M. truncatula*, *P. sativum,* and *T. pratense*. Although these species belong to *Fabaceae*, they differ in their time of evolutionary divergence. *Medicago truncatula*, *T. pratense*, and *P. sativum* are evolutionary closer than *P. vulgaris* and *G. max*. It has been estimated that *T. pratense* and *P. sativum* separated from *M. truncatula* about 25 million years ago, whereas the separation of *P. vulgaris* and *G.* max from *M. truncatula* is expected to date back to 60 million years ago [70]. Therefore, the clustering of the CN, TN, and TNL classes of NLR proteins appears to be congruent with the species diversification in *Fabaceae*.

To sum up, in this study, we carried out genome-wide investigations in nine species of *Fabaceae* to explore the diversity, abundance, and domain architecture of NLR proteins. Our findings revealed several important insights. Firstly, we observed that the abundance of NLR proteins in different species is not correlated with their genome size, suggesting that factors other than genome size contribute to the abundance of these proteins. Secondly, the NB-ARC domain of NLR proteins showed differential preferences for different LRR motifs, with NL1 being the most predominant, followed by NL2 and NL1L2, indicating the presence of three major groups in the NL class of NLR proteins. Thirdly, the domain architecture of NLR proteins exhibited species-specific complexity, with significant variation in the number of unique domains among different species. Notably, the domain architecture of NLR proteins from *M. truncatula* and *T. pratense* displayed more complex domain architectures compared to other species, while *P. vulgaris* exhibited the least complexity. Additionally, diversity analysis using metric multidimensional scaling revealed species-specific clustering in the CN, TN, and CNL classes, which appears to be congruent with species diversification in *Fabaceae*. This study significantly contributes to our understanding of NLR proteins by providing comprehensive insights into their repertoire, domain architecture, diversity, and abundance across nine *Fabaceae* species. These findings offer valuable knowledge about plant defense mechanisms and enhance our understanding of NLR proteins. However, further genome-wide research focusing on resistant and susceptible cultivars of specific plant species is necessary to gain a deeper understanding of plant diseases.

## Funding

This work is partially supported by the Specialty Crop Block Grant AWD00011228 and the 10.13039/100000199USDA Award AWD00011012. This work is also partially supported by the Specialty Crops Research Initiative Award 2018-51181-28420 from the 10.13039/100005825USDA National Institute of Food and Agriculture.

## Data availability statement

The datasets presented in this study can be found in the article/Supplementary Material. The R script used in this work is available in GitHub repository (https://github.com/vishalsnegi/NLR_Fabaceae/blob/main/NLR_fab.R)**.**

## CRediT authorship contribution statement

**Vishal Singh Negi:** Writing – review & editing, Writing – original draft, Methodology, Investigation, Formal analysis, Data curation, Conceptualization. **Rajagopalbabu Srinivasan:** Writing – review & editing, Methodology, Investigation. **Bhabesh Dutta:** Writing – review & editing, Writing – original draft, Visualization, Supervision, Software, Resources, Project administration, Methodology, Investigation, Funding acquisition, Formal analysis, Data curation, Conceptualization.

## Declaration of competing interest

The authors declare that they have no known competing financial interests or personal relationships that could have appeared to influence the work reported in this paper.

## Figures and Tables

**figs1:**
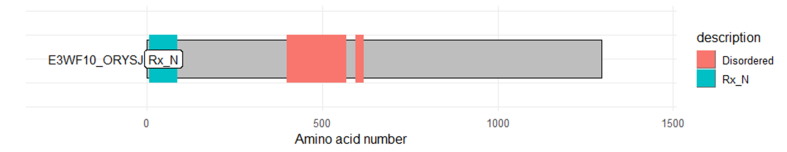


**figs2:**
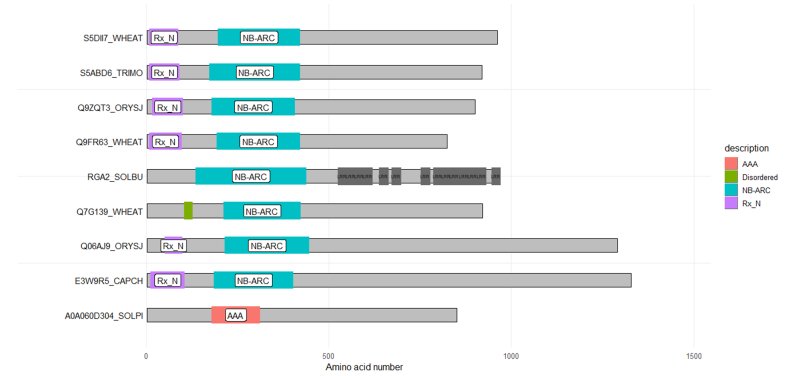


**figs3:**
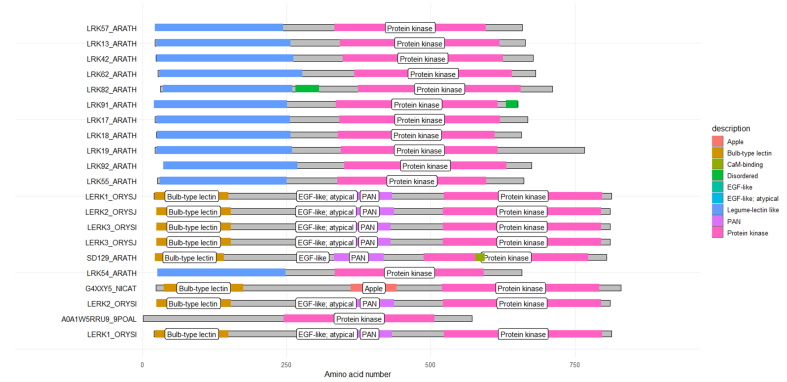


**figs4:**
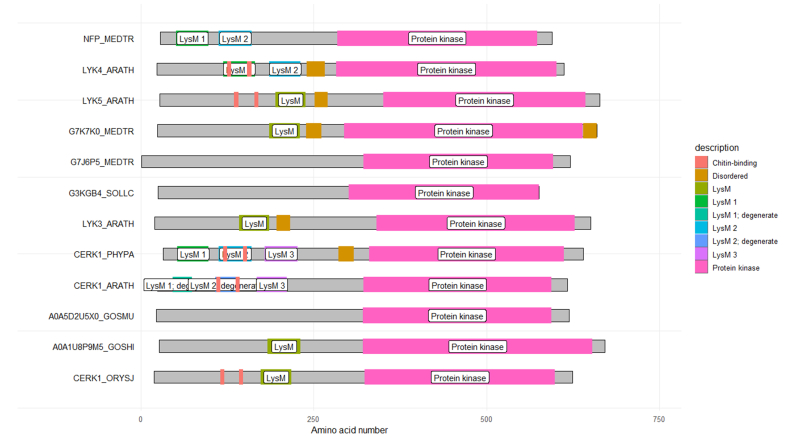


**figs5:**
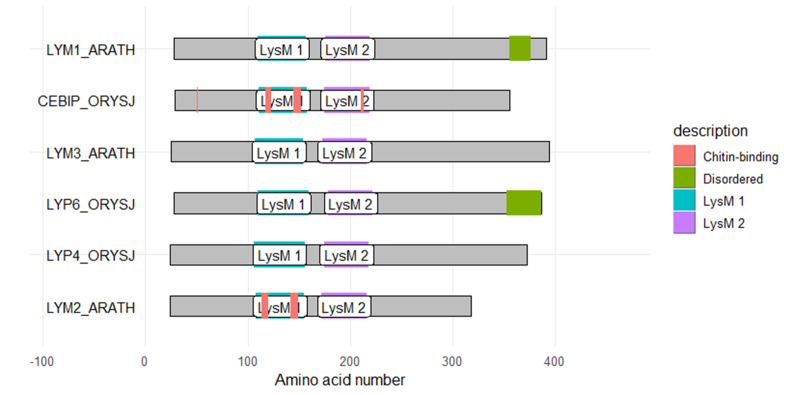


**figs6:**
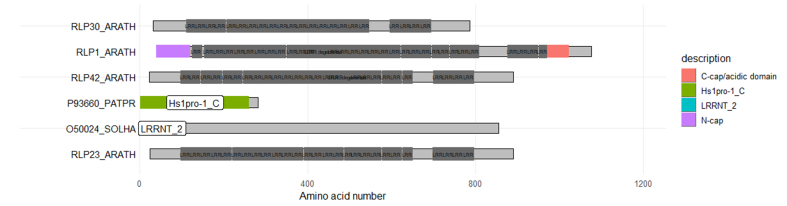


**figs7:**
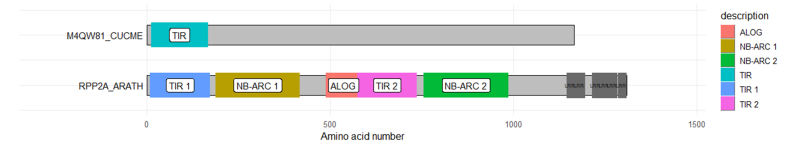


**figs8:**
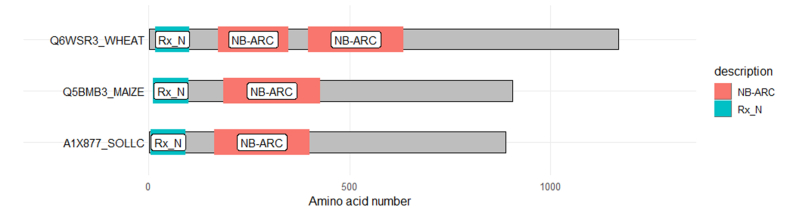


**figs9:**
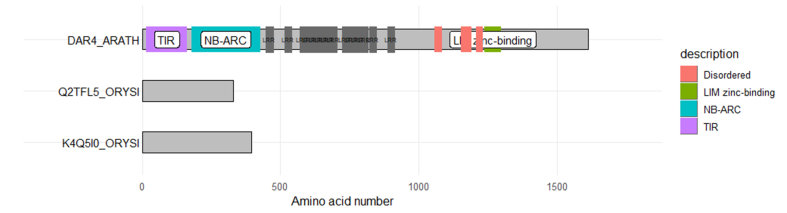


**figs10:**
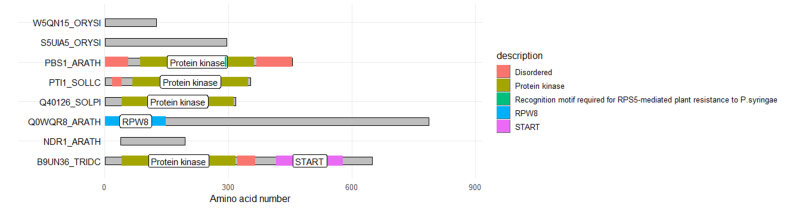


**figs11:**
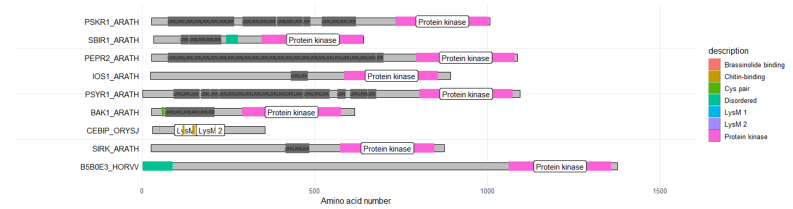


**figs12:**