# Supplementary material for: Diversity, abundance, and domain architecture of plant NLR proteins in Fabaceae
Source: Heliyon. 2024 Jul 12;10(14):e34475. doi: 10.1016/j.heliyon.2024.e34475 (PMC11734081; doi:10.1016/j.heliyon.2024.e34475)
Supplement: Multimedia component 20 [file mmc20.pptx]

## Slide 1
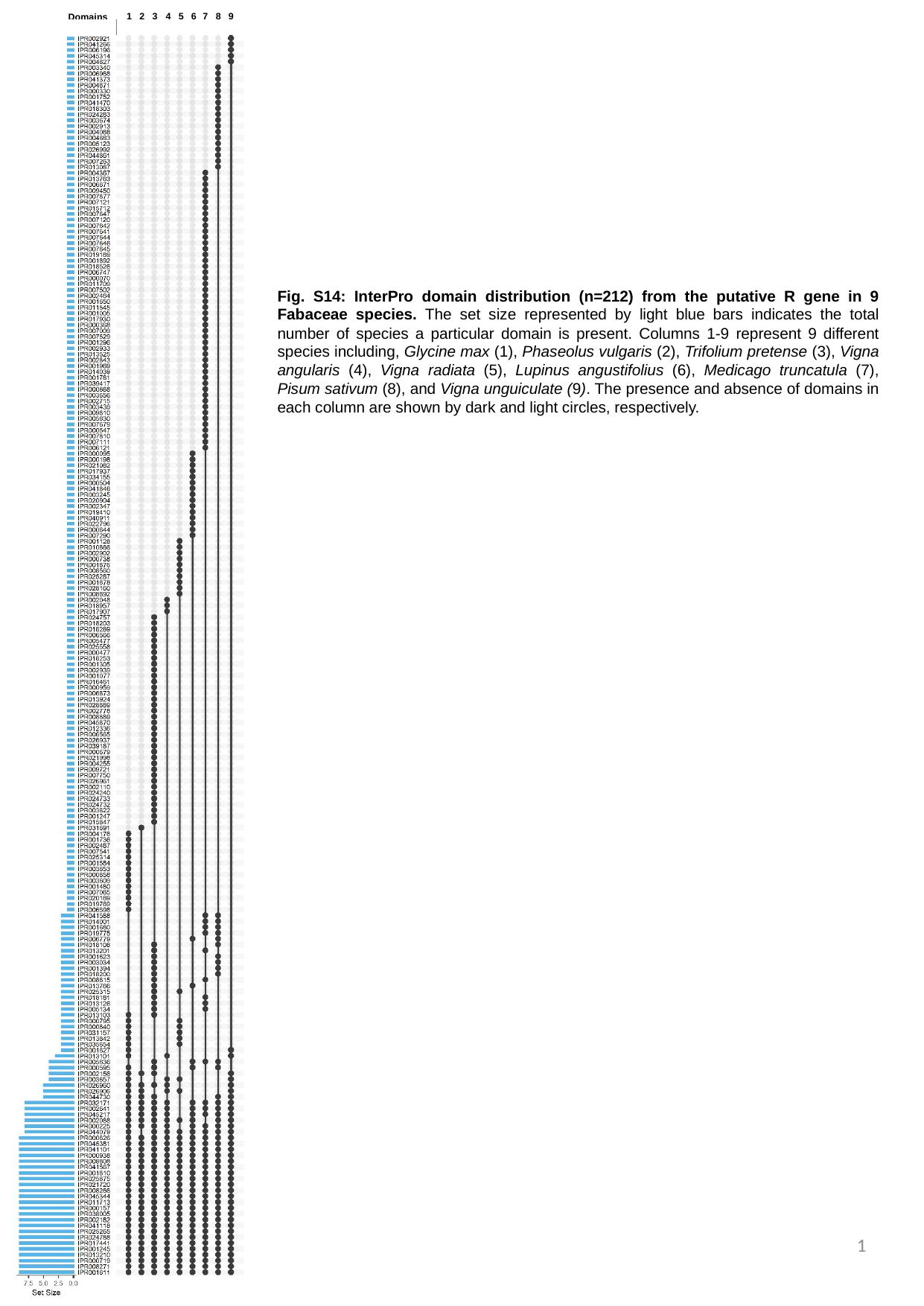

8
9
7
4
6
3
5
2
1
Domains
Fig. S14: InterPro domain distribution (n=212) from the putative R gene in 9 Fabaceae species. The set size represented by light blue bars indicates the total number of species a particular domain is present. Columns 1-9 represent 9 different species including, Glycine max (1), Phaseolus vulgaris (2), Trifolium pretense (3), Vigna angularis (4), Vigna radiata (5), Lupinus angustifolius (6), Medicago truncatula (7), Pisum sativum (8), and Vigna unguiculate (9). The presence and absence of domains in each column are shown by dark and light circles, respectively.
1
