# Supplementary material for: Diversity, abundance, and domain architecture of plant NLR proteins in Fabaceae
Source: Heliyon. 2024 Jul 12;10(14):e34475. doi: 10.1016/j.heliyon.2024.e34475 (PMC11734081; doi:10.1016/j.heliyon.2024.e34475)
Supplement: Multimedia component 21 [file mmc21.pptx]

## Slide 1
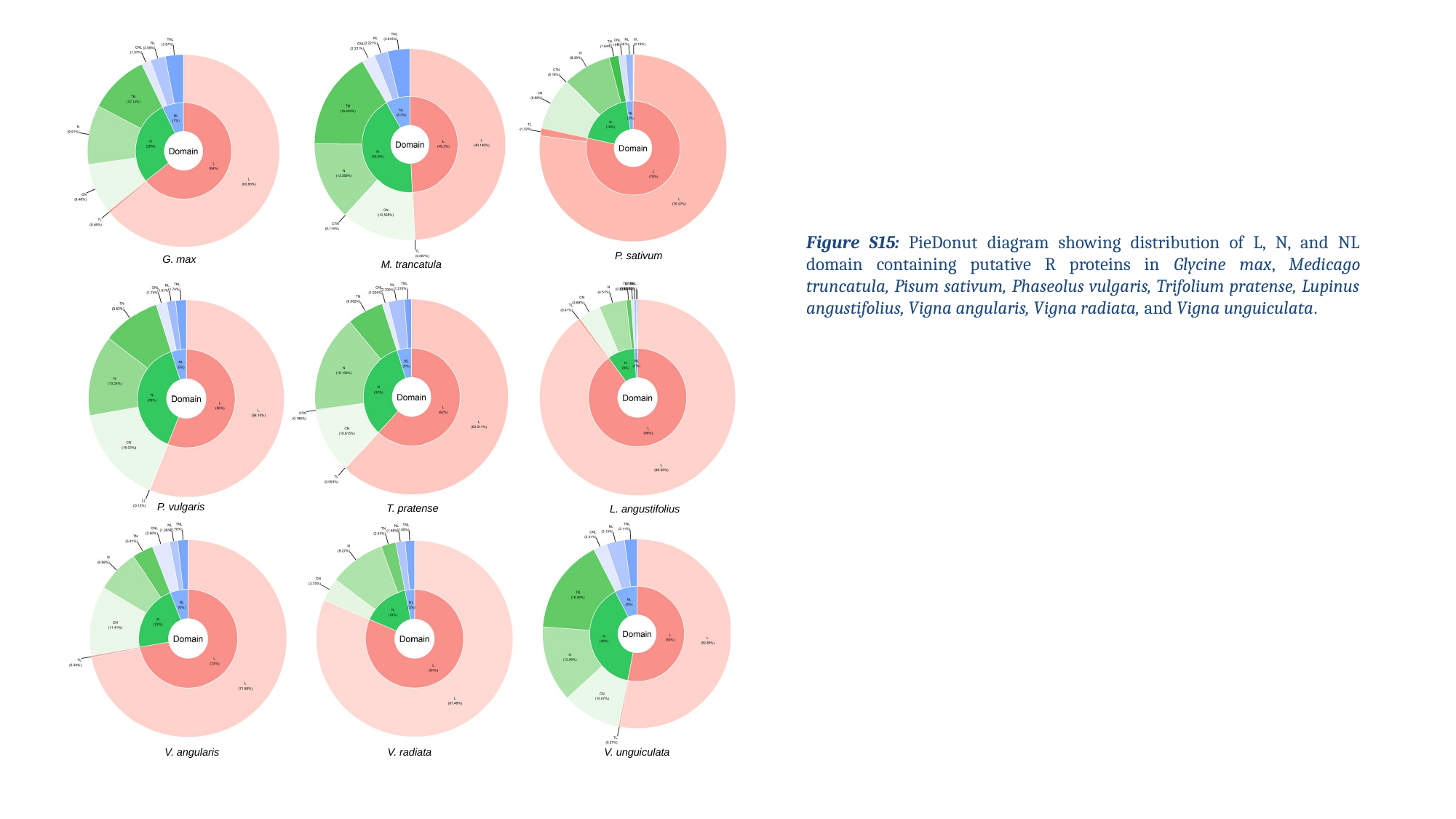

G. max
P. sativum
M. trancatula
L. angustifolius
T. pratense
P. vulgaris
V. unguiculata
V. radiata
V. angularis
Figure S15: PieDonut diagram showing distribution of L, N, and NL domain containing putative R proteins in Glycine max, Medicago truncatula, Pisum sativum, Phaseolus vulgaris, Trifolium pratense, Lupinus angustifolius, Vigna angularis, Vigna radiata, and Vigna unguiculata.
