# Supplementary material for: Diversity, abundance, and domain architecture of plant NLR proteins in Fabaceae
Source: Heliyon. 2024 Jul 12;10(14):e34475. doi: 10.1016/j.heliyon.2024.e34475 (PMC11734081; doi:10.1016/j.heliyon.2024.e34475)
Supplement: Multimedia component 22 [file mmc22.pptx]

## Slide 1
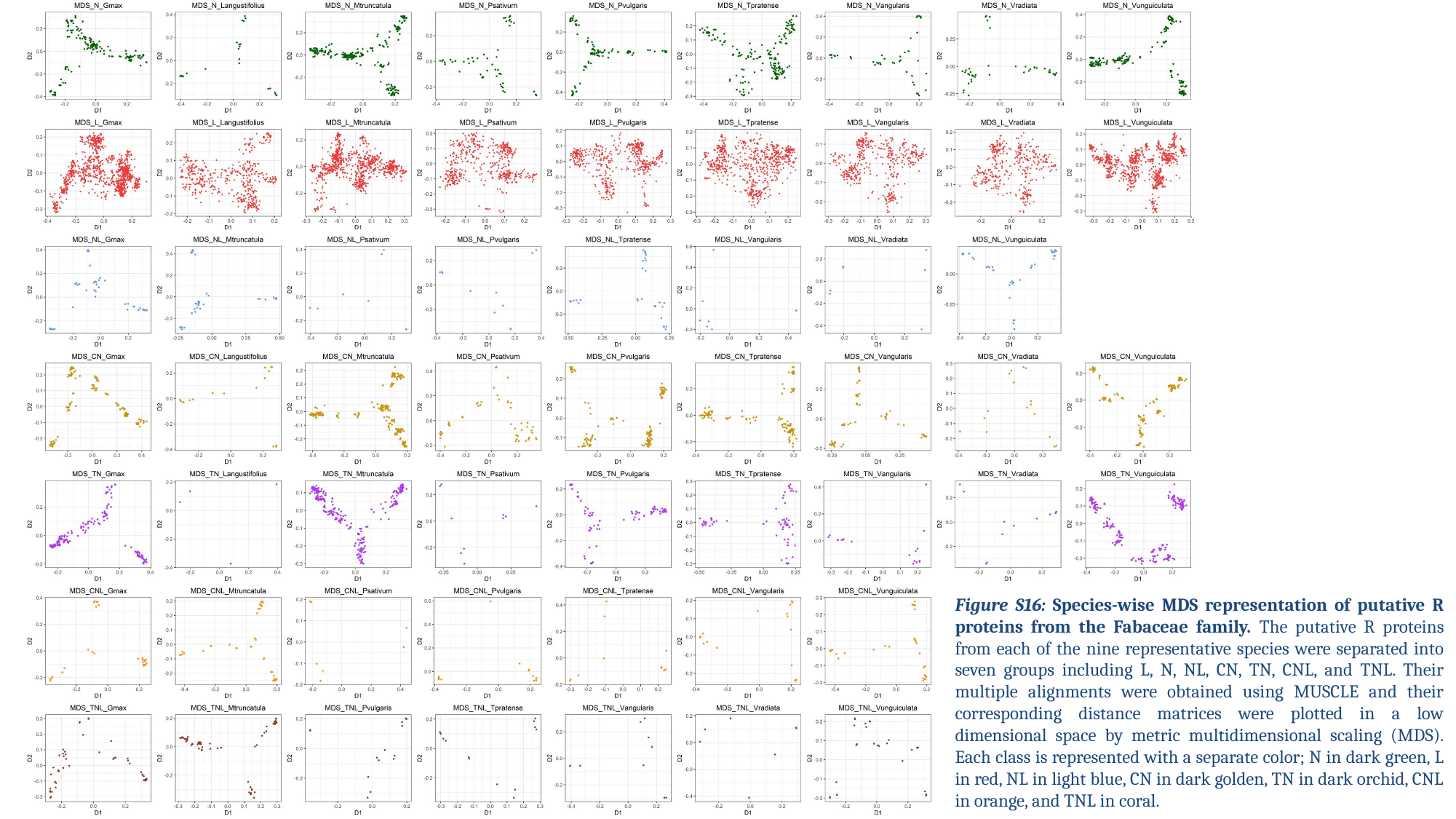

Figure S16: Species-wise MDS representation of putative R proteins from the Fabaceae family. The putative R proteins from each of the nine representative species were separated into seven groups including L, N, NL, CN, TN, CNL, and TNL. Their multiple alignments were obtained using MUSCLE and their corresponding distance matrices were plotted in a low dimensional space by metric multidimensional scaling (MDS). Each class is represented with a separate color; N in dark green, L in red, NL in light blue, CN in dark golden, TN in dark orchid, CNL in orange, and TNL in coral.
